# GlyGen: a knowledgebase linking glycan data with protein and gene data to reveal novel biological connections

**DOI:** 10.21203/rs.3.rs-9982242/v1

**Published:** 2026-07-01

**Authors:** Rene Ranzinger, Robel Kahsay, Urnisha Bhuiyan, Kate Warner, Jeet Vora, Sujeet Kulkarni, Nicole Mathias, Shovan Bhowmik, Vinicius de Souza, K Vijay-Shanker, Maria Martin, Nathan Edwards, Michael Tiemeyer, Raja Mazumder

**Affiliations:** 1. Complex Carbohydrate Research Center, University of Georgia, Athens, GA 30602, USA; 2. Department of Biochemistry and Molecular Medicine, School of Medicine and Health Sciences, The George Washington University, Washington, DC 20007, USA; 3. Department of Biochemistry and Molecular & Cellular Biology, Georgetown University, Washington, DC 20007, USA; 4. Department of Computer and Information Sciences, University of Delaware, Newark, DE 19716, USA; 5. European Molecular Biology Laboratory, European Bioinformatics Institute (EMBL-EBI), Wellcome Genome Campus, Hinxton, Cambridge CB10 1SD, United Kingdom

## Abstract

Glycans influence protein structure and function, modulate tissue development, regulate organ function, drive pathogen interactions, control inflammation and immunity, and impact most disease processes. However, glycan and glycosylation data are frequently difficult to access, existing across heterogeneous resources, with inconsistent representation and limited linkage to the genes, proteins, and protein sites that define their biological context. This fragmentation limits integration with genomics, proteomics, and other omics data, and constrains the discovery of glycan-mediated relationships. GlyGen is a knowledgebase designed to harmonize and integrate glycan, protein, and glycosylation data within a unified, glycosylation-centric data model. The model currently captures 58,841 glycans, 243,702 proteins, and 183,573 glycosylation sites, and extends to motifs, diseases, biomarkers, as well as germline and somatic sequence variations. Data are collected from public resources and literature, standardized using identifiers such as UniProt and GlyTouCan accessions, and integrated through ontology-driven workflows with evidence tracking and attribution. GlyGen supports free access via a web portal, APIs, downloads, and semantic web technologies. The current release (www.glygen.org) integrates diverse datasets across 16 organisms with extensive cross-references and provides an interoperable framework for advancing the integration of glycoscience into the broader biomedical data ecosystem.

## INTRODUCTION

Glycoscience explores the functions, structures, and expression of glycans in living organisms and viruses, aiming to understand their roles in broader molecular and biological contexts. Glycans are typically present as components of glycoconjugates, such as glycoproteins and glycolipids, where they are covalently attached by glycosylation^1^. Glycosylation modulates protein stability, activity, and function, while also influencing protein localization or trafficking in cells and tissues^2^. Glycoconjugates, molecules in which glycans are covalently attached to proteins, or other biomolecules, provide molecular frameworks that facilitate signal transduction, mediate cellular responses, and maintain tissue integrity in health and disease^3^. Many pathogens utilize host glycans as attachment or toxin-binding receptors and, reciprocally, pathogen glycans modulate host immune and inflammatory responses^4^. Glycosylation is dynamically altered in almost all human diseases, especially inflammatory disorders and cancer, where many tumor-associated antigens are specific glycoforms of proteins or lipids^5–7^. In the last two decades, increased clinical awareness has led to the growing identification of primary human disorders caused by defects in glycosylation pathways. These congenital disorders of glycosylation provide important new paradigms for understanding the essential roles of glycan biosynthesis and degradation^8^. The extensive physiological and pathophysiologic importance of glycosylation offers multiple entry points into glycoscience for glycobiologists and other biomedical investigators.

Although data generation related to the roles of glycans and glycosylation across nearly all areas of biology is accelerating rapidly^9–11^ and important advances have been made toward standardization, including the development of glycan identifiers such as GlyTouCan^12^ accessions and representation frameworks such as the Symbol Nomenclature for Glycans (SNFG)^13^, the adoption of these standards across the literature and data resources remains limited and continues to pose challenges for reliable glycan data integration^11^.

Such integration is essential for understanding glycan function, which requires connecting glycan structures to the broader molecular context in which they occur, including the genes and enzymes responsible for glycan biosynthesis, the proteins to which glycans are attached, and the biological systems in which these interactions take place. The absence of harmonized data resources that integrate glycan structures with genomic, proteomic, and functional data has limited the ability to systematically explore glycan knowledge.

GlyGen was launched in 2017 as a knowledgebase that integrates glycan and glycosylation information with protein, gene, genomic, mutational, disease, tissue, and cell-type data. The resource provides open access to these integrated datasets through a web portal, downloadable resources, and programmatic interfaces. Here we describe the architecture, data resources, and analytical capabilities of GlyGen, and present representative use cases that illustrate how integrated glycoscience data can support exploration and hypothesis generation across biomedical research domains.

## RESULTS

### DATA MODEL

**Figure 1.**
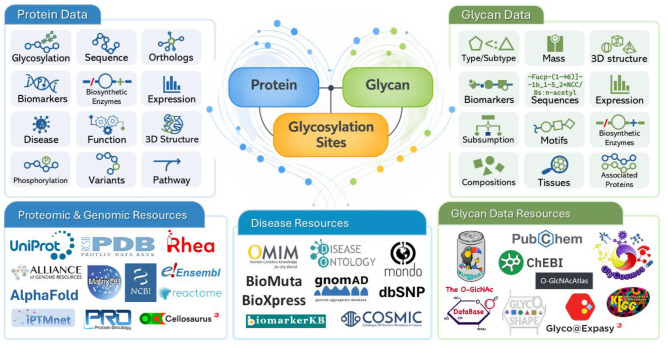
The GlyGen data model components showing the key data attributes in protein and glycan datasets (upper part) alongside selected data sources providing this data. The resources are grouped by the type of data. A full list of the resources integrated and cross-referenced in GlyGen can be found in Supplementary table 2.

The GlyGen data model was initially developed to support the representation of glycan- and protein-centric data, providing a structured framework for organizing core biological entities related to glycans and glycoproteins^14^. Figure 1 illustrates these central concepts, their associated data, and resources providing this data. However, as the scope and diversity of integrated datasets grew over time, the model was progressively extended to accommodate a broader range of biological concepts and annotations. This evolution has enabled the incorporation of additional key concepts, including protein sites, glycan motifs, diseases, publications, and biomarkers, thereby enhancing the model’s ability to capture complex biological relationships and support more comprehensive data integration.

Glycans and glycosylated proteins remain the central focus of the data model, as they represent the primary molecular context of interest. Proteins can carry glycans attached at specific amino acid residues, forming glycoproteins that serve as key integration points. However, the reporting of multiple glycans at a given site reflects both site-specific microheterogeneity and variation across independent studies arising from differences in experimental methods and analytical resolution. At a given glycosylation site, different studies may report distinct glycans or describe them at different levels of structural detail, ranging from simple monosaccharide compositions to fully-defined structures, reflecting differences in analytical resolution and true biological variation across tissues, cell types, or conditions. The model also accommodates proteins without known glycosylation sites that carry other annotations or site-related modifications, and glycans that are not yet linked to a specific protein context. These elements are incorporated to support a more comprehensive and integrated view of glycans and their roles, allowing for future connections between proteins and glycans as new knowledge emerges.

A defining feature of the GlyGen data model is the explicit representation of protein site-level information, which plays a critical role in linking glycosylation to broader contexts, such as genomic mutations. Each protein site corresponds to a defined range within the protein sequence, specified by start and end positions, and acts as an anchor for integrating evidence of glycosylation with related annotations, including germline and somatic mutations, protein structural features, as well as other post-translational modifications (PTMs) occurring within or near glycosylation sequons, the short amino acid sequence motifs that specify potential glycosylation sites. All data are supported by evidence and linked to the relevant literature and original data resource, ensuring transparency and traceability.

### DATA INTEGRATION

GlyGen publishes quarterly releases that incorporate new datasets, annotations, and cross-references. Data integration follows a deliberate strategy designed to ensure completeness and consistency. Rather than simply aggregating glycans and glycoproteins, GlyGen first incorporates a carefully curated reference proteome for each organism. Canonical protein sequences are systematically mapped to all known and predicted isoforms, after which glycan structures associated with that organism are integrated. This approach enables a comprehensive representation of glycosylation knowledge while minimizing redundancy. Newly encountered glycans or glycoprotein annotations are evaluated to determine whether they correspond to existing entries. When appropriate, entries and annotations are merged with existing records rather than creating duplicate entities. As a result, GlyGen serves as a reference resource for organism-specific proteomes and glycan datasets, developed through close collaboration with UniProt^15^ for protein data and with partners in the GlySpace Alliance^16^ for glycan data. Table 1 summarizes the distribution of glycan, glycoprotein, and glycosylation site data in GlyGen, and Supplementary File 1 presents the distribution of proteins and glycoproteins across species. GlyGen, for its core entities, uses widely adopted identifier systems such as UniProt accessions for proteins and GlyTouCan identifiers for glycans. This design ensures compatibility with established community standards and enables seamless integration of annotations from diverse biological databases.

Glycosylation site annotations in GlyGen are aggregated from multiple community resources, direct collaborations with research groups, and literature text mining. In addition to experimentally supported annotations, the resource also provides predicted N- and O-linked glycosylation sites, enabling users to explore potential glycosylation contexts and supporting hypothesis generation for experimental studies. The provenance, processing, and integration of each imported dataset are documented in accompanying BioCompute Objects^17^.

### WEB PORTAL

GlyGen provides multiple complementary access points to the knowledgebase’s underlying data, accessible via human-readable, semantically structured URLs, addressing the needs of both experimental scientists and data-scientists: (1) the GlyGen interactive web portal (https://www.glygen.org), designed for interactive exploration using a web browser; (2) the data site (https://data.glygen.org), which provides harmonized source datasets prior to integration; (3) the RESTful web service API (https://api.glygen.org), which enables programmatic retrieval of GlyGen data in the machine readable JSON format; and (4) the SPARQL endpoint (https://sparql.glygen.org), providing access to the GlyGen content as semantic web triples by the execution of SPARQL queries. While the latter three interfaces primarily support data-science workflows and live data integration with third-party applications, the web portal targets a broad scientific audience, including researchers in glycobiology, proteomics, metabolomics, and related fields who seek to complement wet lab or *in silico* studies with additional glycoscience data.

**Figure 2.**
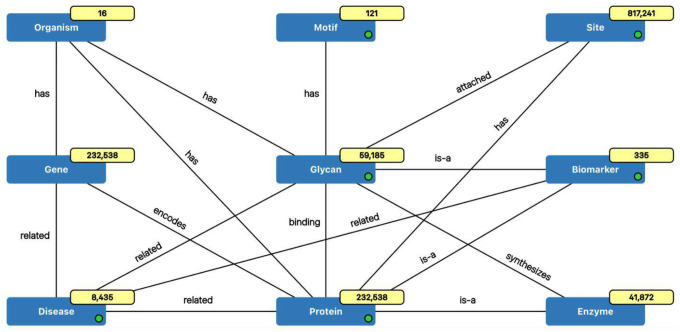
An overview of the major concepts and their relationships in the GlyGen knowledgebase. The total numbers for each concept across the knowledgebase in version 2.10 are shown. Green dots in boxes signify concepts with dedicated information pages in GlyGen, that include a concise summary view that highlights key biological attributes, followed by detailed sections covering annotations, structural data, evidence, and references.

The GlyGen web portal is centered on the unified presentation of diverse biological concepts, including glycans, proteins, glycosylation sites, glycan motifs, biomarkers, diseases, and related annotations (Figure 2, overview of major GlyGen concepts). These data types are integrated into a consistent, searchable framework that emphasizes interoperability and interpretability of biological context within the same web platform. To accommodate different types of users, GlyGen offers several search modes. A global keyword search allows rapid retrieval of relevant entities using gene symbols, protein accessions, glycan identifiers, disease terms, or other free text inputs. In addition, each core concept has a dedicated search page that supports both simple free text queries (simple search) and an extended list of explicit search criteria (advanced search). Search results are presented as tabular lists enriched with key information and a hit score, which can be further refined using concept-specific filters. Hyperlinks connect search results directly to the detailed entity pages, which aggregate all available annotations in a standardized layout. Beyond the traditional search options, GlyGen offers an advanced “Supersearch” interface (https://glygen.org/super-search/) that allows users to query the knowledgebase based on a graphical representation of the key concepts and apply search options to any of these concepts. This search approach enables indirect queries – for example, searching by glycan motif will return not only matching motifs and glycans, but also the glycosylation sites and proteins to which these glycans are attached. Recently, an AI-powered query assistant was introduced, leveraging large language models (LLMs) to translate natural language questions into structured queries for GlyGen API.

The GlyGen “Tools” menu provides data resources and analysis tools, developed to address common user needs beyond exploration of GlyGen’s primary entities. Some tools, such as GlyGen Blast, GlyGen Mapper, GlyGen Batch Retrieval, and GlyGen Isoform Mapper, are tightly integrated into the portal, while others, such as GlycoMotif, GNOme, and GlyGen Sandbox, are maintained as independent subprojects to facilitate rapid development. Supplementary file 2 shows the tools currently available, with a short description of their functionality.

A key design principle of GlyGen front-end is transparent data provenance. From any piece of displayed information, users can directly trace its origin to the original data source or publication, along with the associated supporting evidence.

### GLYGEN-DEVELOPED GLYCOINFORMATICS COMPONENTS

In addition to large-scale data integration and harmonization, this work also addressed critical gaps in the glycoinformatics ecosystem. One such effort is the GlycoMotif data resource, which maintains and curates GlyGen glycan structure motifs (https://glygen.org/list-of-motifs) and their alignments. GlycoMotif organizes the glycan motif lists published by many different groups, in addition to the GlyGen motif collection. Glycan motifs represent recurring substructures with known biological relevance, and GlycoMotif provides a standardized representation and identifiers for motif-based analysis. GlycoMotif also provides GlyGen’s glycan structure classification annotations, using motifs in conjunction with heuristic rules to automatically assign glycan types and subtypes. GlyGen also developed GNOme^18^, an OBO Foundry glycan structure ontology that organizes glycans hierarchically based on subsumption, or degree of characterization. GNOme enables the propagation of annotations from fully-defined glycan structures to monosaccharide compositions, which is used for GlyGen’s glycan classifications, species and tissue annotations, and glycan-motif alignments. To further connect glycan structures with terminology commonly used in the literature, GlyGen developed the Glycan Dictionary^19^, a curated collection of glycan structure terms frequently described as free text in publications. The dictionary provides an important bridge between free text descriptions in the literature and structured glycoinformatics data.

GlyGen also developed the GlycoTree Sandbox to associate glycosylation enzymes with the structures they synthesize. The GlycoTree Sandbox maintains curated super-trees of potential N- and O-linked structures with biosynthetic enzymes from human, mouse, rat, bovine, and pig. Super-tree aligned glycan structures can then be assigned the enzymes responsible for each monosaccharide. These assignments help connect-the-dots between glycosylation enzyme loss and glycan structures, and support efforts to understand how perturbations in glycoenzyme abundance affect specific glycan structures.

Together with GlyGen’s integrated datasets, these resources and tools strengthen the representation and interoperability of glycoscience data and contribute to a more robust foundation for glycoinformatics research.

### BI-DIRECTIONAL DATA EXCHANGE AND QUALITY ASSURANCE

GlyGen facilitates bidirectional data exchange with multiple external resources, including UniProtKB^15^, PubChem^20^, and ChEBI^21^. To date, GlyGen has contributed 1,467,422 cross-references to these resources through bulk dataset submissions. In parallel, GlyGen actively encourages partner resources to link back to its platform, enhancing data accessibility and enabling seamless navigation between shared entries. Because of data sharing and cross-resource exchange, substantial shared content exists between GlyGen and these resources. Specifically, 9,909 glycan entries are also represented in ChEBI, 243,702 protein entries are in common with UniProtKB, and 58,402 glycan entries together with 66,680 protein entries are also represented in PubChem. This strategy ensures that users can access glycoscience data through the resources they already use, while GlyGen remains available for more detailed glycan-centered exploration and analysis. Supplementary File 3 provides a tabular summary of the external data resources integrated into GlyGen.

Beyond data integration, GlyGen applies comprehensive quality control procedures to all incorporated datasets. Identified inconsistencies are systematically reported back to the originating resources to support ongoing correction and curation efforts. To date, datasets and front-end level errors have been identified and communicated to partner resources for resolution. These quality control assessments include, but are not limited to, the detection of amino acid mismatches, discrepancies in PubMed ID references, typographical errors, and annotations of amino acid positions that fall outside valid sequence ranges.

## DISCUSSION

GlyGen supports a wide range of applications for both experimental researchers and data scientists. For many experimental biologists, a typical use case begins with a protein or glycan of interest. Users may search for the protein and navigate to its entry page to examine annotated glycosylation sites, associated glycans, and related biological information such as functional annotations, disease associations, expression patterns, and biomarkers. These pages also provide access to experimentally reported glycosylation data as well as predicted glycosylation sites, which can serve as starting points for experimental validation. Similarly, users interested in glycan structures can explore glycan entry pages to examine structural features, motifs, biosynthetic enzymes, and associated proteins or biological contexts. For bioinformaticians, GlyGen provides additional capabilities that enable large-scale analysis. These include batch retrieval tools, sequence-based queries, advanced search interfaces, APIs, and SPARQL endpoints, allowing users to identify intersections or unions of complex biological features across proteins, glycans, and sites. Data downloads from search results further support downstream computational analysis and integration with external workflows.

The data integration, tool development, and other infrastructure necessary to support GlyGen have led to unexpected capabilities that would otherwise require significant effort to implement. For example, the integration of glycan subsumption and species annotations enables the straightforward generation of a Byonic^22^ glycan database of 374 monosaccharide compositions of N-linked glycan structures annotated as human, with associated GlyTouCan accessions. Web-services for on-demand glycan structure lookup, subsumption alignment, and glycan image rendering support the application of deep-learning-based semantic image interpretation by locating extracted IUPAC sequences in the GlyTouCan accession space and facilitating visual confirmation of correct extraction. Similarly, the use of SVG glycan images with monosaccharide highlighting, developed for the GlycoMotif project, can be used for motifs, enzymes, and other residue level annotations, not just by GlyGen but also by other resources. Finally, the collection of well-described, consistently formatted, glycan data resources derived from internal and external sources in the GlyGen data site (https://data.glygen.org), provides unprecedented access to glycan focused informatics resources in one place. A recent need for publications associated with large-scale N-glycosylation-site analysis in UniProt was quickly satisfied by 16 CSV files (one for each GlyGen supported species) that provide UniProt protein site annotations with the associated PubMed ID.

To illustrate the scope of possible applications and provide an overview of how the resource can be used, the following representative biological use cases are presented.

### Use case: Exploration of Prostate-specific antigen, a glycan-related biomarker.

**Figure 3.**
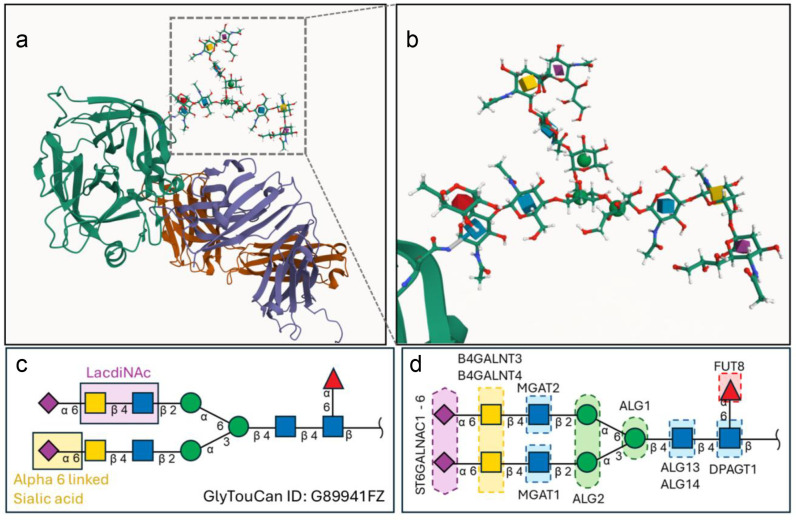
3D structure of PSA (KLK3, UniProt Accession: P07288) with the N-linked glycan G89941FZ at Asn69, with its glycan motifs and the enzymes involved in the biosynthesis. (a) The 3D protein structure of the glycoprotein shows the crystal structure of human PSA complexed with an activating mouse antibody; PSA (green) is shown bound between two Fab fragments (heavy chains, orange; light chains, purple) (PDB ID: https://www.rcsb.org/structure/2ZCH). The 3D glycoprotein structure with the attached glycan was created using the GLYCAM glycoprotein builder (https://glycam.org/). (b) Zoom in on the single N-linked glycan structure attached to Asn69 of PSA. (c) 2D representation of the N-linked glycan (G89941FZ) shown using the SNFG, where monosaccharides are depicted as standardized colored symbols. Highlighted structural elements illustrate glycan motifs with the common motif in the same color. These features have been reported to vary in abundance on PSA glycoforms in prostate cancer and are presented here to contextualize motif-based analysis and glycan-specific annotations within GlyGen. (d) Enzymes involved in the biosynthesis of G89941FZ. For each monosaccharide of the glycan, plausible bioenzymatic enzymes are shown that add the monosaccharide to the glycan. This data is provided by the GlyGen Sandbox (https://sandbox.glyomics.org/explore.html?G89941FZ). If more than one enzyme is listed for a monosaccharide, it means that either of the enzymes may have added the monosaccharide. For example, ST6GALNAC1 – 6 means that ST6GALNAC1, ST6GALNAC2, ST6GALNAC3, ST6GALNAC4, ST6GALNAC5 or ST6GALNAC6 can add the (2–6) linked Neu5Ac residues to the GalNAc residue during biosynthesis of G89941FZ.

The human glycoprotein prostate-specific antigen (PSA, protein name KLK3, P07288) is a leading marker for the detection of prostate cancer (PCa) and for discerning therapeutic efficacy following treatment^23^. KLK3 is a serine-type endopeptidase that is found in circulation either in free form or bound to a serine protease inhibitor (serpin), and the ratio of free to total serum PSA correlates inversely with favorable clinical outcomes^24^. KLK3 possesses a single site for N-linked glycosylation at Asn69. This site is found on the face of the protein opposite to the face that mediates interaction with serpin, thereby suggesting a role for the glycan in establishing which protein domains are available to mediate protein-protein interactions. Molecular modeling emphasizes the spatial restrictions imposed on protein-protein interactions by glycosylation at Asn69 (Figure 3, PDB with glycan modeled). Enhanced biomarker specificity in patient sera has been observed for specific PSA glycoforms^25^. Glycan features associated with increased specificity include the elaboration of LacdiNAc antennae (GalNAcb4GlcNAcb-R) and loss of a6-linked sialic acid (NeuAc), with associated increase of a3-linked NeuAc, both of which are enriched on the Asn69 N-linked glycosylation site in PCa; a weaker association suggests core fucosylation may also enhance specificity^26,27^. (Figure 3, glycan structure features). The GlyGen Protein Details page for PSA (https://glygen.org/protein/P07288) lists 192 N-linked glycan accessions reported at the Asn69 site (Glycosylation Section). The full-screen table viewer permits restriction to fully defined glycans, resulting in sixteen structures, four of which contain the LacdiNAc feature and nine of which are terminally sialylated with α3 or α6-linked NeuAc; evidence badges provide links to the original data-sources for all glycans shown.

The LacdiNAc glycan motif (GGM.000016) is an identified GlyGen motif. The Advanced Glycan Search feature can be used to highlight the glycans of PSA associated with the LacdiNAc motif by entering P07288 in “Glycosylated Protein” and LacdiNAc in “Glycan Motif”, resulting in 15 structures, four of which are fully defined. Two of the fully defined structures have a6-linked sialic acids capping the LacdiNAc antennae and two are core-fucosylated.

Exploring the Glycan Details page of PSA associated LacdiNAc glycan G89941FZ, with sialylated LacdiNAc antennae and core-fucose, provides a list of the biosynthetic enzymes involved in biosynthesis (Biosynthetic Enzymes Section). The GlyGen GlycoTree Sandbox, available using the “Sand Box” icon in the general section at the top of the page, provides a curated list of enzymes for each monosaccharide of the glycan. The SandBox’s Pathways tab provides an interactive pathway builder that summarizes each step of the glycan’s biosynthesis. Enzymes in the Sand Box link back to the corresponding GlyGen Protein Details page for additional information.

The Protein Details page for crucial enzymes involved in the LacdiNAc motif, human B4GALNT3 (Q6L9W6) and B4GALNT4 (Q76KP1), each of which adds the β4-linked GalNAc, indicates that both are expressed in the prostate gland (Expression Tissue Section), and that B4GALNT4 has been significantly associated with prostate cancer (Expression Disease Section). Badges provide links to the source of these assertions, Bgee^28^ and BioXpress^29^, respectively, in this example, allowing users to explore the original data source for their own purposes.

### Use case: Identifying the impact of mutational loss or gain of glycosylation sites.

GlyGen allows analyses that extend beyond inspection of individual glycoprotein or glycan records by integrating protein sequence annotations with genomic variation data at scale. By linking somatic and germline mutations to experimentally validated and predicted glycosylation sites, GlyGen enables systematic identification of glycosylation changes due to loss of glycosylation (LOG) or gain of glycosylation sites (GOG).

An example of a potential disease association with loss of an N-glycosylation site occurs in antithrombin (SERPINC1, P01008)^30^. Mutations affecting the N-glycosylation sequon at Asn135 have been reported to disrupt glycosylation and reduce circulating antithrombin levels, changes that may impact coagulation. Notably, due to differences in protein sequence numbering used across databases and publications, the residue reported as Asn135 in the literature maps to Asn167 in the reference protein reference sequence, which can be readily seen in the GlyGen site detail page (https://glygen.org/Siteview/P01008-1/167). This type of harmonization in GlyGen allows users to correctly identify the affected glycosylation site and integrate sequence variation data with glycosylation annotations. The functional consequences of glycosylation site gain have been demonstrated in other experimental studies, such as the identification of a Thr168Asn missense mutation in IFNGR2 that creates a novel N-glycosylation site and causes Mendelian susceptibility to mycobacterial disease^31^. Subsequent structural analysis almost a decade later showed that the introduced N-linked glycan likely disrupts receptor complex assembly through steric interference, providing a mechanistic explanation for impaired signaling^32^. Integration of data from multiple publications and databases enables exploration of related knowledge in one space (https://glygen.org/Siteview/P38484-1/168).

**Figure 4.**
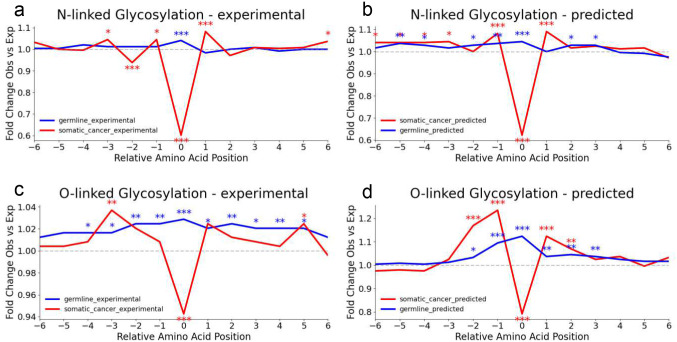
Occurrence of human germline and somatic cancer-associated SNVs around N- and O-linked glycosylation sites. Non-synonymous germline SNVs (blue) and somatic cancer-associated SNVs (red) for +/−6 amino acids (+/− 20 amino acids have a similar profile) relative to the glycosylation site are shown. (a) Experimentally confirmed N-linked glycosylation sites; (b) predicted N-linked glycosylation sites; (c) experimentally confirmed O-linked glycosylation sites; and (d) predicted O-linked glycosylation sites. The Y-axis shows the (observed/expected) fold change of SNV occurrence at each amino acid position on the X-axis. On the X-axis, position 0 denotes the glycosylation site, defined using either experimentally validated glycosylation sites (a, c) or computationally predicted sites (b, d). Statistically significant positions are annotated on the plot as asterisks (*** p < 0.001, ** p < 0.01, * p < 0.05), while the absence of an asterisk denotes lack of statistical support. For N-linked glycosylation sites, statistically significant decreases are accompanied by larger fold changes, whereas for O-linked sites, observed decreases, although statistically significant, show smaller fold changes and are therefore interpreted as more modest effects. In all four plots, the germline data (blue) across all four plots show no notable deviation from expected mutation rates at any amino acid position, including the glycosylation sites. In contrast, the somatic cancer data (red) show markedly lower mutation rate at the glycosylation sites, meaning that fewer mutations are observed than expected at the glycosylation sites. This is consistent with negative selection at the functionally critical residue, suggesting that undisrupted N- and O-glycosylation may have an important role in cancer. In the germline, variation is shaped at the population level and primarily filtered by overall fitness of the individual, such that non-lethal or mildly deleterious mutations at these sites can persist at low frequency and broadly reflect underlying mutational processes. In contrast, tumor evolution is governed by strong selection at the level of individual cells, where glycosylation is critical for protein folding, stability, trafficking, and cell-surface signaling, as well as for immune evasion. Disruption of these sites may therefore impair protein stability or immune recognition, resulting in negative selection against such mutations. Under this model, glycosylation-site residues would appear relatively unconstrained in germline variation but selectively preserved in cancer. Furthermore, the somatic cancer data with predicted O-linked glycosylation sites display more fluctuation across positions (d) when compared with the experimental O-linked glycosylation sites (c), suggesting the difficulty of accurately predicting O-linked sites.

Beyond individual cases, computational proteome-wide analyses have demonstrated that loss and gain of N-linked glycosylation sites due to sequence variation are widespread^33^ (Supplementary file 4, Supplementary file 5). Integrated GlyGen data reveal that N-linked glycosylation sequons tend to exhibit reduced somatic sequence variability relative to their surrounding regions, consistent with selective constraints on maintaining these functionally important sites, as shown in Figure 4. In contrast, germline variation does not show a comparable depletion at these sites, suggesting that population-level variants largely reflect mutations that have already been filtered for compatibility with protein function and are therefore tolerated at rates similar to surrounding regions. GlyGen integrates this data and implements these concepts by providing methods to query, filter, and download such data across the proteome, showing how data integration can help identify candidates for downstream experimental and functional investigation, because for most such variants, the biomedical effects remain unknown.

### Use case: Linking datasets: UniProt to GlyGen to PubChem.

Erythropoietin (EPO) is a glycoprotein hormone produced mainly by the kidneys to stimulate red blood cell production in the bone marrow in response to low oxygen levels in the blood. Some synthetic analogs of EPO, collectively known as erythropoiesis-stimulating agents (ESAs), have been engineered with structural or glycosylation modifications to prolong duration of action and improve pharmacokinetic stability in the treatment of anaemia^34^. In this use case, a researcher is investigating how the glycosylation of human EPO (P01588) and the chemical features of its attached glycans contribute to its stability and pharmacokinetic properties. As in most glycobiology queries, this research requires exploring data across diverse disciplines, in this case proteomics, glycomics, and biochemistry.

Supplementary file 6 (figure) demonstrates how users starting on the UniProtKB/SwissProt entry page for the human EPO glycoprotein (https://www.uniprot.org/uniprotkb/P01588) can explore detailed curated proteomic data for EPO, and follow links from the basic glycosylation site data in the PTM/processing section of UniProt, to the detailed glycosylation section of the EPO GlyGen protein page (https://glygen.org/protein/P01588#Glycosylation). The GlyGen glycosylation table provides comprehensive information on experimentally reported, predicted, and literature-mined glycosylation sites. For example, the glycan G25392ZR has been reported on several EPO amino acid sites and is a fully sialylated tetra-antennary glycan, meaning that it may contribute to increasing the biological half-life and stability of EPO^34^. In the glycosylation table and anywhere on the protein page, selecting a GlyTouCan ID, in this case G25392ZR, links to the corresponding glycan page in GlyGen (https://glygen.org/glycan/G25392ZR), where the user can explore detailed glycan-specific information such as names, 3D view, associated proteins, organism, tissue, and glycan biosynthesis. For additional glycan information, GlyGen provides cross-references to many different specialist resources, including chemical repositories such as PubChem (https://pubchem.ncbi.nlm.nih.gov/compound/91852997), where the researcher can look at the glycan’s chemical features, which may contribute to the molecular stability of EPO.

### CONCLUSIONS AND FUTURE DIRECTIONS

Future development of GlyGen will focus on expanding both the depth of knowledge and the breadth of integrative capabilities across the biomedical data ecosystem. A major priority is the continued incorporation of information from the scientific literature using advanced natural language processing (NLP) and large language model (LLM) based approaches. Building on current text-mining pipelines in GlyGen, we will develop semi-automated workflows in which text mining-driven extraction of glycosylation events, glycan structures, enzymes, and biological context is combined with a curator-in-the-loop framework. This approach will enable scalable ingestion of high-quality, evidence-linked data while maintaining the accuracy and interpretability required for a reference knowledgebase. In parallel, GlyGen will expand its predictive capabilities. Current efforts focused on N-linked and mucin-type O-linked glycosylation will be extended to additional glycosylation types such as O-GlcNAcylation, alongside the integration of emerging machine learning methods for glycan analysis from mass spectrometry data^35^. Another key direction is the ongoing development of richer knowledge graph representations that connect GlyGen data with other biomedical knowledge graphs spanning genomics, proteomics, metabolomics, and disease biology^36^. By embedding glycosylation-aware relationships into these interconnected frameworks, GlyGen will help reveal biological associations that are not apparent within isolated datasets and support systems-level analyses. We will continue to strengthen collaborations with model organism databases, UniProt, PubChem, ChEBI, worldwide glycoinformatics resources via GlySpace Alliance, and ontology development efforts to formalize and standardize concepts of glycan function. Building on our ongoing discussions within the community^37^, these collaborations will support the development of shared vocabularies and computable representations of glycan-mediated biological processes. Collectively, these directions will further position GlyGen as a central resource for integrating glycoscience with mainstream biomedical research.

## ONLINE METHODS

### DATA

#### Data Collection

GlyGen integrates glycan-, protein-, and glycoprotein-centric data from major public repositories and specialized research datasets through a standardized, ontology-driven workflow. To provide a broader biological context, the resource also incorporates complementary information, including genetic variants and mutations, disease associations, phosphorylation and other post-translational modifications, biomarkers, glycan–protein interactions, and structural data. At the beginning of each release cycle, GlyGen performs coordinated downloads from external resources to obtain the most current datasets. The retrieved files are subjected to quality checks to confirm successful downloads and to verify that file structures and formats remain intact. Downloaded datasets are compared against the previous release, and any significant increases or reductions in data volume are investigated to confirm they reflect intentional changes by the source resource. When source dataset formats change or new collaborations introduce additional resources, the corresponding data processing workflows are updated to ensure continued compatibility.

#### Protein sequences

Protein sequences are obtained from reference proteome datasets in UniProt, with isoforms mapped to canonical sequences using genomic coordinates to ensure a non-redundant representation. This approach yields a curated and consolidated set of canonical proteins. For example, the human reference proteome (UP000005640) in UniProtKB contains 83,526 entries (release 2026_01), whereas after isoform consolidation in GlyGen, this is reduced to 20,663 canonical proteins in the reference proteome.

#### Glycan sequences

Glycans are categorized based on the degree of structural information available, forming a hierarchy that reflects increasing levels of detail and confidence. At the highest level, fully defined glycans have complete structural characterization, including monosaccharide identities, linkage positions, and anomeric configurations. Incomplete glycans retain some of this structural information but lack certain details, such as specific linkages or stereochemistry, reflecting limitations in experimental resolution. Moving further down, topology-level glycans describe the overall connectivity and branching of monosaccharides without specifying exact linkage positions or configurations, capturing the general scaffold of the structure. At the lowest level, composition-level glycans provide only the counts and types of constituent monosaccharides (e.g., Hex_5_HexNAc_2_) without any information on their arrangement or connectivity. Together, these categories enable GlyGen to integrate glycan data from diverse experimental sources while accommodating varying levels of structural ambiguity, with fully defined structures offering the highest specificity and composition-level data providing the broadest coverage.

#### Text mining

Text mining is employed to systematically extract glycosylation-related information from the scientific literature and augment GlyGen annotations. An initial approach leveraged natural language processing and rule-based methods, building on prior systems such as RLIMS-P^38^, to capture glycoprotein context, glycan features, experimental details, and residue-level site information from publications. This framework enabled structured extraction of glycan-protein relationships and associated metadata for integration with existing resources. Subsequently, an automated machine learning-based pipeline, GlycoSiteMiner, was developed to specifically identify experimentally validated, sequence-specific glycosylation sites from PubMed abstracts. By combining entity recognition with filtering algorithms to reduce false positives, this approach addressed challenges associated with free-text reporting of sites lacking standardized protein identifiers. The pipeline is applied to PubMed abstracts.

#### Creating harmonized and standardized GlyGen datasets

Data collected from various resources is used to create protein-, glycan-, and glycoprotein-centric processed datasets. To ensure consistency across integrated resources, GlyGen relies on authoritative identifiers for core biological entities. For example, proteins are referenced using canonical accessions from UniProtKB, while glycans are indexed using identifiers assigned by GlyTouCan. When alternative or legacy identifiers are present, they are mapped to their canonical forms to maintain uniform entity representation and reduce redundancy. Each GlyGen dataset created in this step undergoes rigorous validation and quality-control procedures, which include verification of file structure, confirmation of primary identifiers, validation of residue or amino-acid coordinates where applicable, and additional manual evaluation to ensure biological accuracy and data integrity.

#### Statistics and Cross-references of Data Available from Front-end and used in this manuscript

Using this integration approach, glycosylation data have been assembled across multiple organisms, with human annotations representing the largest component. For humans, GlyGen includes 20,663 proteins, of which 14,142 are annotated glycoproteins, representing approximately 68% of the human proteins represented in the resource. These proteins collectively contain 226,986 curated glycosylation sites. In terms of N-linked glycosylation, sequence analysis indicates that 15,443 of human proteins contain the N-linked glycosylation sequon (N-X-S/T), and GlyGen captures 4,042 of these proteins through experimentally supported annotations, making it one of the most comprehensive integrated resources for human glycoprotein information.

Beyond human, GlyGen integrates glycosylation data across multiple organisms, including mouse (21,803 proteins; 10,964 glycoproteins of which 3,604 are predicted), bovine (26,939 proteins; 5,937 glycoproteins including 5,773 are predicted), rat (22,403 proteins; 6,569 glycoproteins of which 5,177 are predicted), pig (22,804 proteins; 4,690 glycoproteins of which 4,640 are predicted), chicken (18,372 proteins; 4,015 glycoproteins of which 3,985 are predicted), hamster (23,887 proteins; 3,162 predicted glycoproteins), and zebrafish (26,744 proteins; 6,090 glycoproteins of which 4,682 are predicted). Additional model systems include fruit fly (13,822 proteins; 3,179 glycoproteins of which 2,849 are predicted), yeast (6,065 proteins; 1,372 glycoproteins of which 870 are predicted), *Arabidopsis* (27,448 proteins; 4,558 glycoproteins of which 4,266 are predicted), and *Dictyostelium* (12,718 proteins; 1,989 glycoproteins of which 1,986 are predicted). Viral glycoproteins, including those from SARS-CoV-2, SARS-CoV, and hepatitis C virus, are also represented. In parallel with protein annotations, GlyGen catalogs large collections of glycan structures across species, including 25,088 glycans associated with human data, and thousands more reported in mouse (9,733), bovine (9,517), rat (9,028), and other organisms (23,644). Table 1 summarizes the glycan, glycoprotein, and glycosylation site data in GlyGen.

For protein entries, GlyGen provides links to a wide range of widely used molecular and biomedical resources. These include protein and sequence repositories such as UniProtKB and RefSeq, structural resources including PDB, AlphaFoldDB, and iCn3D, and functional annotation resources such as InterPro, Pfam, PANTHER, and CDD. Additional connections link proteins to gene-centric resources (HGNC, GeneID, GeneCards), pathway databases (Reactome, KEGG), protein interaction and immune databases (IntAct, IEDB), and disease and phenotype resources including OMIM, HPO, and BioMuta. These links allow users to seamlessly navigate from GlyGen protein entries to related structural, functional, and biomedical data. GlyGen glycans are assigned GlyTouCan identifiers, enabling interoperability with the broader glycoscience ecosystem. Additional cross-references link glycans to chemical and glycan databases such as PubChem and ChEBI maintained through active collaborations. As part of the GlySpace Alliance, GlyGen also exchanges and harmonizes data with major glycoinformatics resources GlyConnect^39^ and GlyCosmos^40^, enabling coordinated glycan representation and integration across the glycoscience data ecosystem.

#### Using BioCompute Objects for evidence tracking

Dataset BioCompute Objects (BCOs) are generated in accordance with the current BCO specification (biocomputeobject.org). Each BCO is constructed from the perspective of the data processing workflow, capturing detailed metadata for every processing step applied to the dataset. Serving as a comprehensive “readme,” these BCOs provide a transparent record of how the dataset was generated, enabling a clear understanding of its provenance, processing, and usage conditions. By standardizing this metadata, BCOs facilitate proper attribution, define licensing terms, support workflow sharing among researchers, and enhance reproducibility. All dataset BCOs are stored in machine-readable JSON format and are accessible for viewing and download via the GlyGen data portal (https://data.glygen.org).

#### Analysis of germline and somatic SNVs around glycosylation sites

The germline mutation data (i.e., somatic status = 0) contains data from the EBI variant API mapped to the canonical proteins in GlyGen, and the somatic cancer data contains all variants from the BioMuta Database, which contains nonsynonymous SNVs associated with cancer mapped to GlyGen canonical proteins. Full details on how these datasets were generated can be found in their BioCompute Objects (https:/data.glygen.org/GLY_001534 for the human germline mutations dataset and https:/data.glygen.org/GLY_001537 for the human cancer mutations dataset).

The integration pipeline for the SNV data consists of three main steps to analyze the relationship between glycosylation sites and genetic variants. First, glycosylation site and variant datasets are extracted from human protein mutation and glycosylation files, producing consolidated datasets of experimental and predicted glycosylation sites that have missense variants. Second, these datasets are mapped by identifying variant-site pairs where variants occur within +/−20 amino acids of a glycosylation site and assigning each variant a relative position with respect to that site, generating mapping files stratified by glycosylation type (e.g., N-linked or O-linked), variant type (e.g., somatic or germline), and site evidence (experimental or predicted). Third, for each relative position (41 total positions from −20 to +20), a two-sided binomial test is applied using scipy.stats.binomtest to assess whether the observed number of variants deviates from random expectation (p = 1/41), producing output files of positions −6 to +6 that report counts, total variants, expected probability, and p-values, thereby highlighting positions with significant enrichment or depletion of mutations around glycosylation sites (Figure 4). The scripts and entire pipeline are available as a Google Colab Python notebook on GitHub (https://github.com/glygener/colab-notebooks), which, when executed, generates the plots using the proteoform and mutation datasets available in the latest GlyGen release. Using the above method, applied to data-release v-2.10.1, 10,778,683 germline mutations and 3,647,974 somatic cancer mutations were identified. We extracted 7,211 experimentally verified N-glycosylation sites, 6,369 predicted N-glycosylation sites, 17,993 experimentally verified O-glycosylation sites, and 5,751 predicted O-glycosylation sites impacted by SNVs. This analysis illustrates an example of the data mining and analysis which is enabled by the multiple resources data integrated in GlyGen.

### BACKEND

#### Docstore database and APIs

Using data structures or models developed to represent protein, site, glycan, disease and biomarker records, processed GlyGen datasets are used to create JSON objects stored within a MongoDB (https://www.mongodb.com/) document store database. This docstore serves as the backend for a range of GlyGen web services as well as external applications. Programmatic access to these data objects is provided through the GlyGen API (https://api.glygen.org), which is fully documented using Swagger (https://swagger.io/). While some web services offer general functionalities such as searching, listing, and retrieving detailed records from the MongoDB docstore, others are specifically tailored to address particular biological questions or use cases identified by the GlyGen user community.

#### Triplestore database and SPARQL

All data in GlyGen docstore is also available in Resource Description Framework (RDF) format, utilizing namespaces from established ontologies. Protein-centric information is represented using the UniProt Core Ontology (https://doi.org/10.1038/npre.2009.3193.1), while glycan-centric data are described through the GlycoRDF Ontology^41^. Glycoprotein data are modeled using a combination of the GlyGen Ontology and the Glycoconjugate Ontology (https://github.com/glycoinfo/GlycoCoO), with glycoprotein instances when possible linked to corresponding entries in the Protein Ontology (PRO)^42^. The triples generated using these ontologies are stored in Virtuoso (https://vos.openlinksw.com/owiki/wiki/VOS) triplestore database.

These triples are also accessible through SPARQL queries available via the GlyGen front-end, enabling flexible and programmatic data retrieval. An ontology knowledge graph can also be explored interactively using WebVOWL, providing a visual representation of relationships within the data (https://sparql.glygen.org/webvowl/#glygen). Endpoint URIs are provided to support direct querying, and an OWL file describing the ontology is available to clarify the underlying logic of the triples. This ontology is updated as new data becomes available, ensuring it reflects the most current knowledge captured in GlyGen. Additionally, users can download the complete set of triple stores as a compressed archive of N-Triples (NT) files for local use and analysis.

### FRONTEND

GlyGen Frontend uses JavaScript library React version 19.1.1 to develop user interfaces with a focus on reusable components. The Material UI Bootstrap library is used to develop clean, modern components, search interfaces, and information pages. GlyGen has integrated the Nightingale library^43^ to display protein site annotations, glycosylation data and glycan structures. Mol* library^44^ is used to display 3-D glycan and protein structures. Glycoglyph^45^ a glycan structure drawing tool, is integrated and used to generate GlycoCT sequences, which are then used to perform structure search.

### AI ASSISTED SEARCH

GlyGen AI Query Assistant provides a user-friendly interface for entering natural-language queries about glycans or proteins. The query is sent to the AI Assistant API, which uses a predefined system prompt and Open AI’s low-latency, cost-efficient GPT-4o mini model to convert the query into an advanced-search API compatible JSON. The advanced-search API then uses this mapped JSON to retrieve data from the GlyGen knowledgebase and present the results to the user.

## Supplementary Material

1

## Figures and Tables

**Table 1.** Glycoproteomic and glycomic data content in GlyGen. Table summary of the distribution of glycan, glycoprotein, and glycosylation site data available in GlyGen. The glycan section is organized by structure definition level, encompassing a spectrum from fully defined glycans, with complete structural and linkage information, to compositions that specify only monosaccharide constituents without connectivity. Glycan types and subtypes describe the structural features of the glycans, whereas types and subtypes in the glycoprotein and site sections represent protein glycosylation types. The glycoprotein and site sections are further categorized by glycosylation evidence type: reported, literature-mined, and predicted. Reported entries correspond to glycosylation data supported by experimental studies or publications. Literature-mined entries are derived from automated extraction of published literature using NLP and LLMs and subsequently verified through human curation. Predicted entries indicate glycosylation sites inferred by computational prediction models. Abbreviations used in the table include GPI anchor (glycosylphosphatidylinositol anchor). Metadata from the GlyGen 2.10.1 release cycle and future release cycles are archived and accessible at https://data.glygen.org/ln2downloads/tmp/paper/.

Category	Type/Subtype	Count			
**A. Glycan Structures**		**Glycan Structure Definition Level**			
		*Fully Defined*	*Incomplete*	*Topology*	*Composition*
	N-Linked Glycan	3,646	13,642	6,777	3,612
	O-Linked Glycan	1,900	4,179	2,916	1,669
	Glycosphingolipids	727	1,807	1,548	758
	GPI Anchors	3	8	8	4
	Glycosaminoglycans	126	590	496	215
	Human Milk Oligosaccharides	416	912	373	144
	C-Linked Glycan	1	2	5	3
**B. Glycoproteins**		**Glycosylation Evidence**			
		*Reported*	*Literature Mined*		*Predicted*
	N-Linked Glycosylation	8,418	480		14,251
	O-GlcNAcylation	18,156	82		212
	O-GalNAcylation	194	21		199
	O-Mannosylation	3	0		17
	O-Fucosylation	21	1		25
	O-Glucosylation	67	1		11
	O-Galactosylation	17	0		31
	C-Mannosylation	20	2		23
**C. Glycosylation Sites**		**Glycosylation Evidence**			
		*Reported*	*Literature Mined*		*Predicted*
	N-Linked Glycosylation	20,227	951		49,381
	O-GlcNAcylation	41,866	137		342
	O-GalNAcylation	741	39		1,516
	O-Mannosylation	35	0		69
	O-Fucosylation	77	1		86
	O-Glucosylation	234	2		58
	O-Galactosylation	47	0		75
	C-Mannosylation	70	4		90

## Data Availability

All GlyGen data are freely available at GlyGen data portal (http://data.glygen.org) and are distributed under the Creative Commons Attribution 4.0 International License (CC BY 4.0), allowing unrestricted use, distribution, and reuse with appropriate attribution. Versioned datasets are maintained and remain accessible independently of the availability of the portal, triplestore, or APIs, ensuring continued access to released data. All software developed and maintained by the GlyGen team, including frontend and backend components, APIs, and data integration and processing pipelines, is openly available through the GlyGen GitHub organization (https://github.com/glygener) under permissive open-source licenses (GPL-3.0 or MIT, as specified per repository). Data are also shared with GlySpace partner resources, and this open and distributed model supports long-term sustainability, transparency, reproducibility, and interoperability within the broader bioinformatics ecosystem.
