# Supplementary material for: GlyGen: a knowledgebase linking glycan data with protein and gene data to reveal novel biological connections
Source: Res Sq. 2026 Jul 1:rs.3.rs-9982242. Preprint. [Version 1] doi: 10.21203/rs.3.rs-9982242/v1 (PMC13345530; doi:10.21203/rs.3.rs-9982242/v1)
Supplement: 1 [file NIHPPRS9982242V1-supplement-1.pdf]

SUPPLEMENTARY FILES

Supplementary file 1. The distribution of proteins and glycoproteins in GlyGen by species. Inner segments represent the total number of proteins per species. Outer segments show the proportional breakdown within each species into glycoproteins (GP) - annotated from experimental, predicted, and/or literature-mined evidence - and non-glycoproteins (NGP). Bovine, Arabidopsis and Zebrafish have the largest proportion of total proteins in GlyGen, each accounting for around 11% of all proteins. Human has the largest number of glycoproteins (14141) for any species in our datasets, representing a 21% share of all glycoproteins, and it also has the highest percentage of glycoproteins within an individual proteome making up 68% of the Human protein dataset. The viruses SARS-CoV-2 (17 proteins including 4 glycoproteins), HCoV-SARS (15 proteins including 5 glycoproteins), HCV-H77, and HCV-Japanese are also present in the dataset; however, their proportions are too small to be visually represented in the chart. HCV-H77 and HCV-Japanese each contain a single polyprotein which includes a glycoprotein.

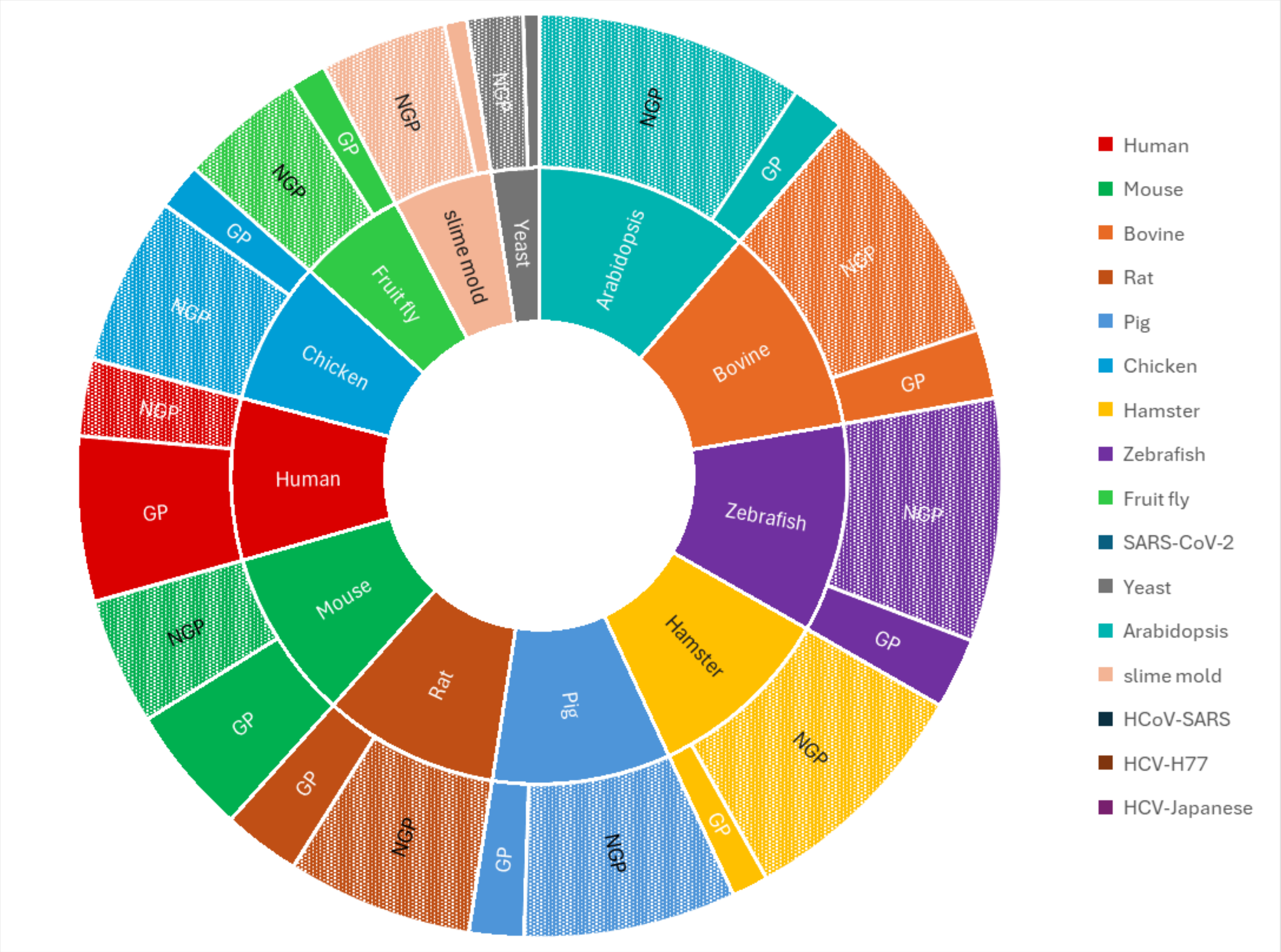

Supplementary file 2. Overview of tools available through the Tools menu of the GlyGen web portal. The table summarizes the major GlyGen tools for discovery, visualization, and curation, including resources for lookup of GlyTouCan IDs, glycan sequence conversion, identifier mapping, batch data retrieval, glycan image generation, and community data submission. For each tool, the name, a short description, and a direct access URL are provided.

Tool Name	Description	URL
**Glycan**		
Glycan Sequence Lookup	Lookup the accession for a glycan sequence in WURCS, GlycoCT, IUPAC, or monosaccharide composition format.	https://glylookup.glyomics.org/
Glycan Structure Dictionary	List of terms for glycans used in glycobiology manuscripts.	https://wiki.glygen.org/Glycan_structure_dictionary
GlycoMotif Wiki	GlycoMotif data-resource for GlyGen glycan motifs, glycan classifications, and their alignments to glycan structures.	https://glycomotif.glyomics.org/
GlyGen Sandbox	GlyGen Sandbox data-resource for GlyGen curated biosynthetic enzymes, and their alignments to glycan structures.	https://sandbox.glyomics.org/
Structure Browser	Interactive graphical browser for GNOme subsumption ontology glycans.	https://gnome.glyomics.org/
Glycan Image Extractor	Extract glycan structure semantics from glycobiology manuscript figures.	https://extractor.glyomics.org/
Glymage	Generate interactive (and non-interactive) SNFG glycan images from a glycan accession or sequence, on demand.	https://glymage.glyomics.org/
Sequence Format Converter	Convert a glycan accession or sequence to specific glycan sequence formats.	https://converter.glyomics.org/
**Protein**		
GlyGen BLAST	Search protein sequences against GlyGen proteins using the BLAST sequence search engine.	https://www.glygen.org/blast/
GlyGen Batch Retrieval	Retrieve protein information for a list of accessions in tabular form, with user-specified columns and filtering criteria.	https://www.glygen.org/batch-retrieval/
GlyGen Isoform Mapper	Map protein loci from the GlyGen canonical accession to UniProt isoform accessions.	https://www.glygen.org/isoform-mapper/

Tool Name	Description	URL
**Glycan and Protein**		
GlyGen Mapper	Map protein and glycan ids from one accession space to another.	https://www.glygen.org/mapper/
GlyTableMaker	Store and share glycan and glycoprotein data, with appropriate metadata, for inclusion in GlyGen.	https://glygen.ccrc.uga.edu/tablemaker/

Supplementary file 3. Tabular summary of the external data resources integrated into GlyGen, including the number of entries displayed from each resource, whether reciprocal cross-references are provided, update frequency (per release, packaged from an alternative source, or last updated date), and data type classification (protein, glycan, or supplemental). In total, GlyGen integrates data from 71 unique resources, spanning diverse domains of glycobiology, proteomics, genomics, and structural biology. If different data types (e.g., glycan, protein) are integrated from a resource, a row for each data type with corresponding numbers is present. Some resources also retrieve data directly from GlyGen, including PubChem, ChEBI, RefSeq, NCBI Gene, Flybase, Europe PMC, and GlyTouCan, where we provide 1,467,422 cross-references (in total) as bulk submission datasets formatted specifically for integration into each respective resource. GlyGen is updated on a quarterly release cycle, during which existing datasets are refreshed, and new resources are incorporated to expand data coverage.

The following resources are referred to using abbreviations: Alliance of Genome Resources (AGR), Bacterial Carbohydrate Structure Database (BCSDB), Bgee gene expression database (Bgee), Conserved Domain Database (CDD), Consortium for Functional Glycomics (CFG), Chemical Entities of Biological Interest (ChEBI), Chemical Database of Bioactive Molecules (ChEMBL), Digital Object Identifier (DOI), Ensembl Genome Browser (ENSEMBL), Glycan-Associated Disease Resource (GADR), Glyco-Disease Literature and Pathway Atlas (GDLPA), GLYCAM molecular modeling tools (GLYCAM), Glycan Naming and Subsumption Ontology (GNOme), Glycan Structure Dictionary (GSD), HUGO Gene Nomenclature Committee (HGNC), Human Phenotype Ontology (HPO), Immune Epitope Database (IEDB), The O-GlcNAc Database - Medical College of Wisconsin (MCW), Mouse Genome Informatics (MGI), Mendelian Inheritance in Man (MIM), Monarch Disease Ontology (MONDO), Metabolomics Workbench (MW), Orthologous MAtrix (OMA), Protein Data Bank (PDB), Protein Families Database (PFAM), NIH Illuminating the Druggable Genome resource (PHAROS), PRoteomics IDEntifications Database (PRIDE), Protein Ontology (PRO), Research Collaboratory for Structural Bioinformatics Protein Data Bank (RCSB PDB), Rat Genome Database (RGD), Saccharomyces Genome Database (SGD), The Arabidopsis Information Resource (TAIR), Uber Anatomy Ontology (UBERON), Virus Pathogen Resource (ViPR), and Zebrafish Information Network (ZFIN).

Resource Name	Xrefs from GlyGen to resource (Count)	Reciprocal Xrefs	Update Frequency	Data Type
AGR	105,620	No	Packaged from EBI	Protein
AlphaFold	165,301	No	Packaged from EBI	Protein
Araport	23,896	No	Packaged from EBI	Protein
BCSDB	735	No	Per Release	Glycan
Bgee	128,320	No	Packaged from EBI	Protein
BiomarkerKB	317	Yes	Per Release	Protein
BiomarkerKB	18	Yes	Per Release	Glycan
BioMuta	16,745	Yes	Per Release	Protein
BioXpress	445,694	No	Per Release	Protein

BRENDA	8,864	No	Packaged from EBI	Protein
CarbBank	34,154	No	Last Updated: 11/03/2022	Glycan
CAZy	2,663	No	Packaged from EBI	Protein
CDD	154,091	No	Packaged from EBI	Protein
CFG	5,432	No	Last Updated: 09/01/2020	Glycan
ChEBI	9,921	Yes	Per Release	Glycan
ChEMBL	7,853	No	Packaged from EBI	Protein
dictyBase	12,180	No	Packaged from EBI	Protein
Disease Ontology	7,467	No	Per Release	Supplemental
DOI	46	No	Per Release	Supplemental
ENSEMBL	61,437	No	Packaged from EBI	Protein
euHCVdb	7	No	Packaged from EBI	Protein
FlyBase	13,973	No	Packaged from EBI	Protein
GADR	1,104	No	Per Release	Glycan
GDLPA	18,911	No	Last Updated: 06/06/2022	Protein
GeneCards	20,573	Yes	Packaged from EBI	Protein
GeneID	190,976	No	Packaged from EBI	Protein
Genomics England	100	No	Per Release	Protein
GLYCAM	5,366	No	Per Release	Glycan
GlyConnect	3,145	Yes	Per Release	Protein
GlyConnect	5,145	Yes	Per Release	Glycan
GlycoEpitope	147	No	Per Release	Glycan
GlycoProtDB	4,196	No	Last Updated: 01/21/2021	Glycan
Glycosciences.de	12,866	No	Per Release	Glycan
GlyCosmos	58,404	Yes	Per Release	Glycan
GSD	138	Yes	Per Release	Glycan
GlycoShape	1,782	In Process	Per Release	Glycan
GlyTouCan	58,841	Yes	Per Release	Glycan
GNOme	58,598	Yes	Per Release	Glycan
GPTwiki	101	Yes	Per Release	Glycan
HepatitisCOnline	2	No	Packaged from EBI	Protein
HGNC	20,474	No	Packaged from EBI	Protein
HPO	5,446	No	Last Updated: 05/25/2025	Supplemental

iCn3D	195,190	No	Packaged from EBI	Protein
IEDB	705,572	No	Packaged from EBI	Protein
IntAct	55,334	No	Packaged from EBI	Protein
InterPro	953,541	No	Packaged from EBI	Protein
IPTMnet	43,340	Yes	Per Release	Protein
KEGG Pathway	4,872	No	Packaged from EBI	Glycan
KEGG Pathway	167,493	No	Per Release	Protein
MassIVE	58,724	No	Packaged from EBI	Protein
MatrixDB	13	No	Per Release	Glycan
MCW	17,832	Yes	Per Release	Protein
MGI	21,970	No	Packaged from EBI	Protein
MIM	23,869	No	Packaged from EBI	Protein
MONDO	10,201	No	Per Release	Supplemental
MW	30	No	Last Updated: 09/10/2024	Glycan
NCBI Taxonomy	20	No	Per Release	Supplemental
O-GlcNAc Atlas	6,012	No	Per Release	Protein
OMA	162,796	No	Per Release	Protein
OrthoDB	278,650	No	Packaged from EBI	Protein
Panther	418,000	No	Packaged from EBI	Protein
PDB	8,116	No	Per Release	Glycan
PDB	195,190	No	Packaged from EBI	Protein
PFAM	354,478	No	Packaged from EBI	Protein
PHAROS	19,992	Yes	Last Updated: 03/19/2020	Protein
PRIDE	1,048	No	Packaged from EBI	Protein
PRO	81,172	No	Packaged from EBI	Protein
PubChem	74,142	Yes	Per Release	Glycan
PubMed	317,463	No	Per Release	Supplemental
RCSB PDB	1,073	No	Per Release	Protein
RCSB PDB	1,387	No	Per Release	Glycan
Reactome	212,305	No	Packaged from EBI	Protein
Reactome	63	No	Per Release	Glycan
RefSeq	182,566	No	Per Release	Protein
RGD	24,316	No	Packaged from EBI	Protein
Rhea	347	No	Packaged from EBI	Glycan

Rhea	87,441	No	Packaged from EBI	Protein
Sandbox	30,146	Yes	Per Release	Glycan
SGD	6,069	No	Packaged from EBI	Protein
GlyTableMaker	14,112	Yes	Per Release	Glycan
TAIR	27,786	No	Packaged from EBI	Protein
UBERON	148	No	Per Release	Supplemental
UniCarbDB	842	No	Per Release	Glycan
UniCarbKB	1,674	No	Last Updated: 1/11/2022	Protein
UniCarbKB	796	No	Last Updated: 1/11/2022	Glycan
UniProtKB	1,001,114	No	Per Release	Protein
ViPR	2	No	Packaged from EBI	Protein
Viral Glycome	16	No	Last Updated: 08/24/2023	Protein
ZFIN	20,222	No	Packaged from EBI	Protein

Supplementary file 4. Summary of mutations in GlyGen that impact glycosylation where each unique mutation is defined by the combination of protein accession, site position, and amino acid substitution. In GlyGen, mutations associated with an impact on glycosylation are sequence variants that are predicted to alter protein glycosylation. These include the gain or loss of an N-glycosylation sequon, as well as the loss of an O-glycosylation site. The table reports the total number of mutations with annotated impact on glycosylation and highlights the subset linked to glycosylation changes across different organisms. Mutation types represented in the dataset include missense, frameshift, stop-gained, in-frame deletions and insertions, as well as other variant classes. Mutation data are available for multiple organisms and vary in composition by species. For rat, the dataset consists of 149 germline mutations, all of which are missense variants. For mouse, a total of 6,753 germline mutations is included, comprising 6,695 missense variants and 58 stop-gained variants. Mutation datasets for human include somatic and germline mutations that impact glycosylation from UniProtKB (https://www.uniprot.org/) and cancer mutation data from the BioMuta database (https://hive.biochemistry.gwu.edu/biomuta). The human datasets contain a broader spectrum of mutation types, including 169,029 missense variants, 732 frameshift variants, 696 stop-gained variants, 86 in-frame deletions, 8 insertions, and 60 variants of other mutation classes. Not all human mutations, particularly those derived from cancer mutation datasets, result in a gain or loss of glycosylation. In total, GlyGen includes 348,729 human mutations across all variant types.

	**Total Mutations Impacting Glycosylation**	**Gain of N-glycosylation site**	**Loss of N-glycosylation site**	**Loss of O-glycosylation site**
Human	170,611	97,330 (57%)	41,067 (24%)	32,214 (19%)
Mouse	6,753	4,118 (61%)	1,859 (28%)	776 (11%)
Rat	149	111 (75%)	18 (12%)	20 (13%)

Supplementary file 5. Distribution of disease annotations for glycosylation affecting mutations in human. Panels (a–f) depict the distribution of disease annotations across three classes of glycosylation effects (gain of N-glycosylation and loss of N- or O-glycosylation) derived from the GlyGen germline and cancer mutation datasets. The germline and cancer datasets capture complementary aspects of human sequence variation and comprise data retrieved from both UniProtKB and BioMuta, respectively. This includes 10,778,683 germline entries and 3,647,974 cancer-related entries. The germline dataset represents the majority of unique mutations and reflects a broad spectrum of inherited genetic variation; however, only a small fraction (~4%) of these variants currently have associated disease annotations. The relatively low proportion of disease-annotated variants reflects both the inherent rarity of experimentally or clinically validated disease-associated mutations and the stringent curation criteria applied by upstream resources such as UniProtKB, which prioritize high-confidence disease associations. In addition, GlyGen retains only variants classed as pathogenic to affect glycosylation sites for these datasets, further reducing the subset of disease-associated mutations captured in this analysis. In contrast, the cancer mutation dataset is inherently disease-linked, with nearly all entries associated with specific cancer types, making it the primary contributor to disease-annotated mutations in GlyGen. Panels (a,b) correspond to mutations associated with a gain of N-glycosylation sequons, panels (c,d) represent loss of N-glycosylation sequons, and panels (e,f) represent loss of O-glycosylation sites. For each category of glycosylation impact, panels on the left (a, c, e) illustrate the distribution of cancer-associated mutations, derived from the GlyGen cancer mutation dataset. Within this group, the majority of annotations are concentrated in skin cancers (13–22%) and gastrointestinal cancers (21–29%), indicating consistent representation of these cancer types across all classes of impact on glycosylation in GlyGen. Panels on the right (b, d, f) show the distribution of non-cancer disease annotations and cancer risk–associated mutations, derived from the GlyGen germline mutation dataset. In contrast to the cancer panels, these distributions are dominated by hereditary (24–32%) and developmental (28–29%) disease categories, with relatively smaller contributions from other disease classes. The proportion of mutations annotated with glycosylation impact is consistent across datasets when stratified by this impact, with N-glycosylation gain (~0.7–0.9%), N-glycosylation loss (~0.28–0.34%), and O-glycosylation loss (~0.86–1.21%) observed at comparable frequencies across cancer and germline mutation datasets. These results show the current status of the disease annotation distribution in the GlyGen germline and cancer datasets and serve to illustrate one of several types of analyses that may be possible with data harnessed by GlyGen.

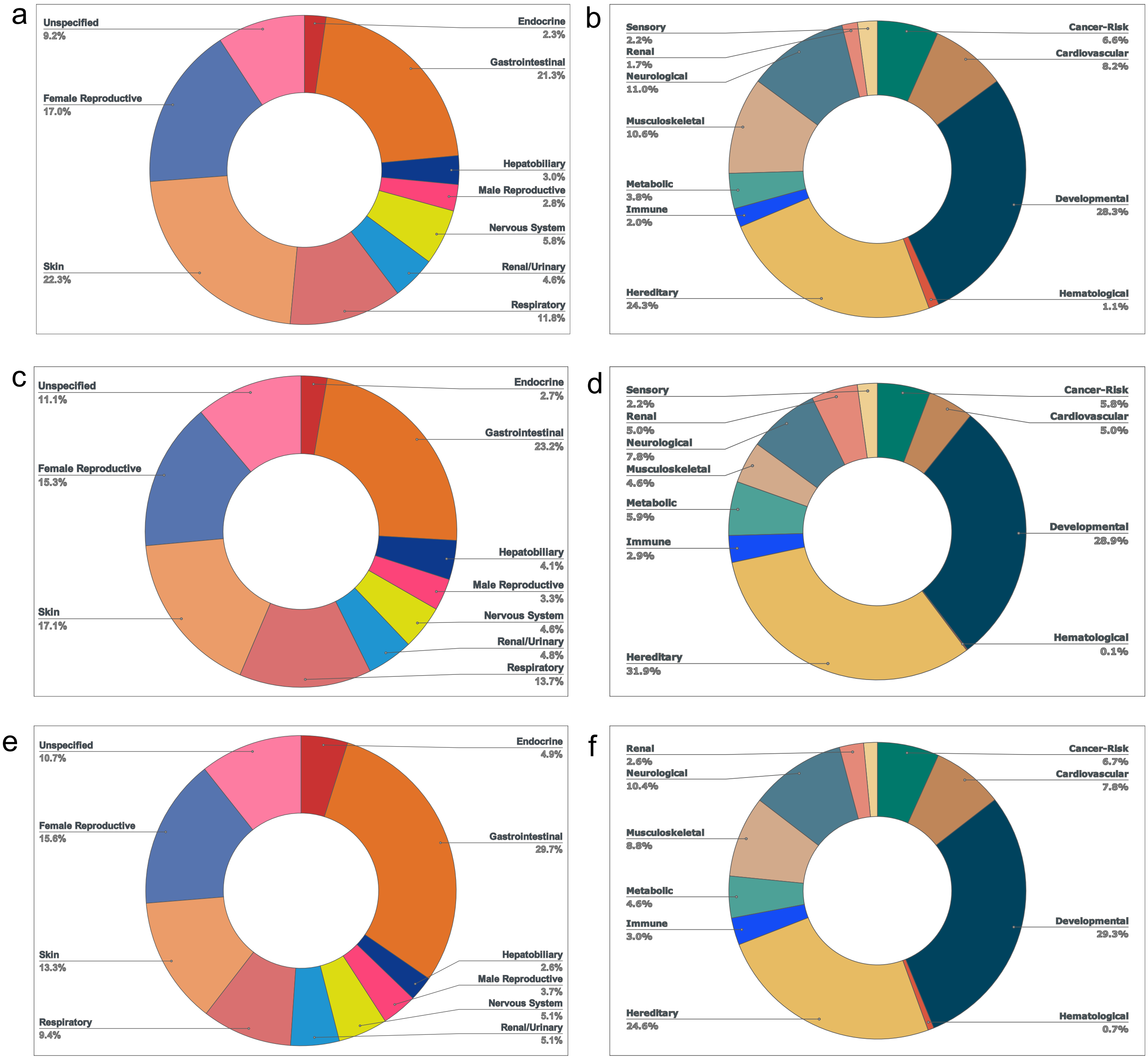

a
Unspecified 9.2%
Endocrine 2.3%
Gastrointestinal 21.3%
Female Reproductive 17.0%
Hepatobiliary 3.0%
Male Reproductive 2.8%
Nervous System 5.8%
Skin 22.3%
Renal/Urinary 4.6%
Respiratory 11.8%
b
Sensory 2.2%
Renal 1.7%
Neurological 11.0%
Cancer-Risk 6.6%
Cardiovascular 8.2%
Musculoskeletal 10.6%
Metabolic 3.8%
Immune 2.0%
Developmental 28.3%
Hereditary 24.3%
Hematological 1.1%
c
Unspecified 11.1%
Endocrine 2.7%
Gastrointestinal 23.2%
Female Reproductive 15.3%
Hepatobiliary 4.1%
Male Reproductive 3.3%
Skin 17.1%
Nervous System 4.6%
Renal/Urinary 4.8%
Respiratory 13.7%
d
Sensory 2.2%
Renal 5.0%
Neurological 7.8%
Musculoskeletal 4.6%
Cancer-Risk 5.8%
Cardiovascular 5.0%
Metabolic 5.9%
Immune 2.9%
Developmental 28.9%
Hematological 0.1%
Hereditary 31.9%
e
Unspecified 10.7%
Endocrine 4.9%
Female Reproductive 15.6%
Gastrointestinal 29.7%
Skin 13.3%
Hepatobiliary 2.6%
Male Reproductive 3.7%
Nervous System 5.1%
Respiratory 9.4%
Renal/Urinary 5.1%
f
Renal 2.6%
Neurological 10.4%
Cancer-Risk 6.7%
Cardiovascular 7.8%
Musculoskeletal 8.8%
Metabolic 4.6%
Immune 3.0%
Developmental 29.3%
Hereditary 24.6%
Hematological 0.7%

Supplementary file 6. A use case to investigate how glycosylation of human Erythropoietin EPO affects its molecular stability and pharmacokinetic properties using the links between UniProt, GlyGen, and PubChem. (a) The UniProt page for human EPO (P01588) includes manually curated and automatically annotated information on protein function, sequence features, 3D structures, interactions, subcellular location, variants, disease, and expression. Glycosylation data is found in the "PTM/processing section", which provides the three N-linked and one O-linked glycosylation site positions, amino acids, the type of linkages, and, when available, the nature of the reducing terminal sugars. Additional data can be found under the "PTM Databases" where the user can find the summarized glycosylation data from GlyGen including the number of identified individual glycans and their type, in this case 190 identified N-linked glycans on three sites, and 18 identified O-linked glycans on one site (as of UniProt Release 2026_01), as well as a link to the corresponding glycoproteomic data in GlyGen. Selecting the GlyGen link takes the user directly to the glycosylation section of EPO on the GlyGen protein page. (b) The glycosylation section of the GlyGen protein page contains a detailed list of glycosylation sites, reported glycans attached to the protein, predicted sites, and sites identified from text mining publications. GlyGen provides data on the four EPO glycosylation sites and lists the 159 N-linked glycans reported at three sites, the 17 O-linked glycans reported at one site, and the 101 glycans identified as associated with the protein, but the exact site is unknown (GlyGen release 2.10). Selecting the full screen view enables the user to restrict the list of glycans and/or sites. In this use case, the researcher would be most interested in fully sialylated tetra-antennary glycans that can increase the biological half-life and stability of EPO. Filters can be used to reduce the table to only fully-defined glycans that contain the sialic acids NeuAc and NeuGc, to find glycans of interest such as G25392ZR, which occurs on multiple sites on the EPO protein. In the table, the GlyTouCan ID of the glycan links to the glycan page in GlyGen. (c) On the glycan page the user can explore glycan-specific information such as associated proteins, glycan expression and enzymes involved in the glycan's biosynthesis. In the Cross-references section, cross-references to many different resources are provided, including chemical repositories such as PubChem. These cross-references are hyperlinks connecting to the corresponding information pages in the referenced database. (d) On the PubChem compound page, the researcher can find chemical-level properties of the glycan, such as the chemical structure, the number of carboxyl groups (negative charge), hydrogen bonding capacity, and structural bulk.

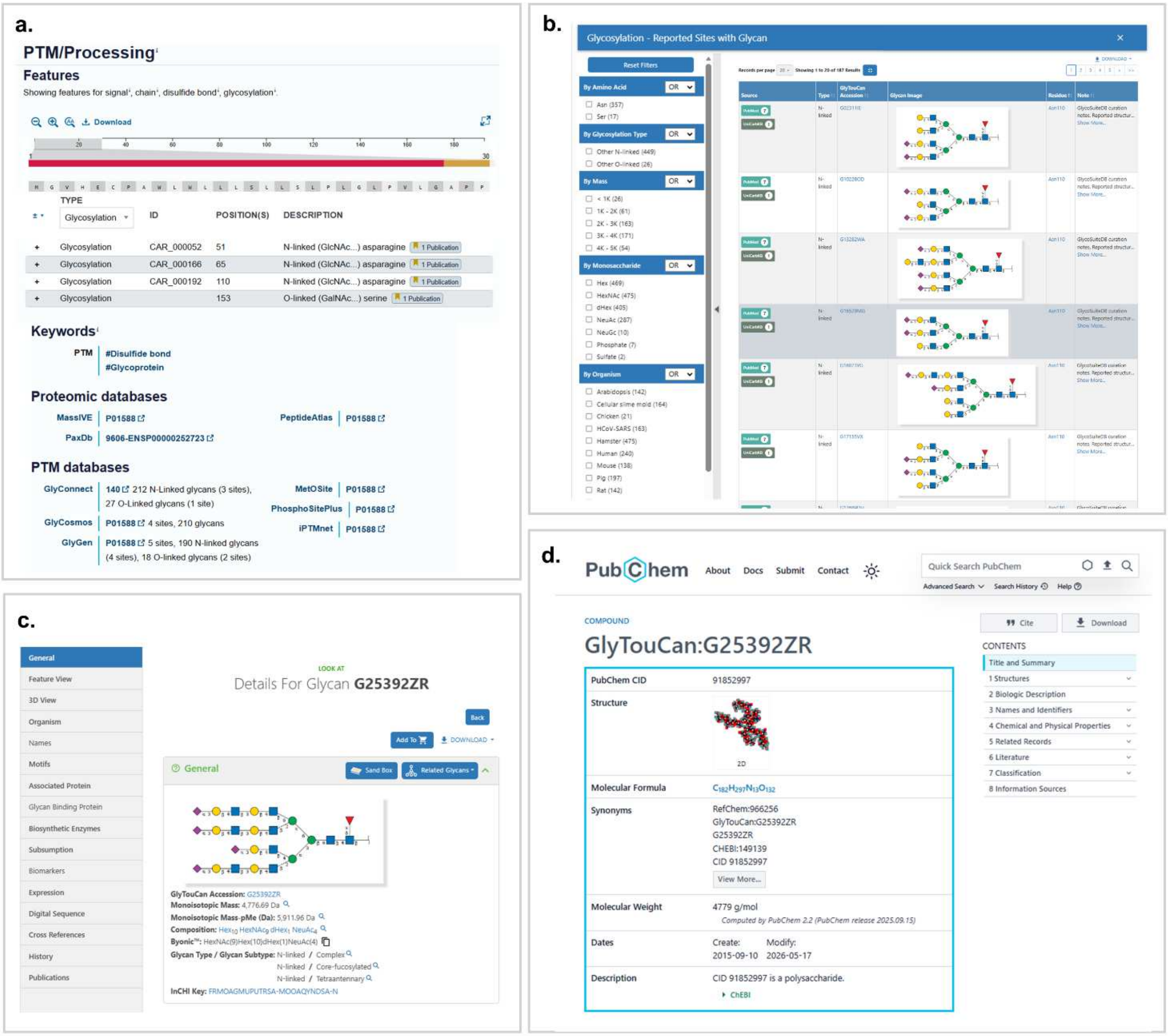
a.
PTM/Processing
Features
Showing features for signal, chain, disulfide bond, glycosylation.
Download
TYPE
Glycosylation
ID
POSITION(S)
DESCRIPTION
Glycosylation CAR_000052 51 N-linked (GlcNAc...) asparagine 1 Publication
Glycosylation CAR_000166 65 N-linked (GlcNAc...) asparagine 1 Publication
Glycosylation CAR_000192 110 N-linked (GlcNAc...) asparagine 1 Publication
Glycosylation 153 O-linked (GalNAc...) serine 1 Publication
Keywords
PTM #Disulfide bond #Glycoprotein
Proteomic databases
MassIVE P01588
PeptideAtlas P01588
PaxDb 9606-ENSP00000252723
PTM databases
GlyConnect 140 212 N-Linked glycans (3 sites), 27 O-Linked glycans (1 site)
MetOSite P01588
PhosphoSitePlus P01588
GlyCosmos P01588 4 sites, 210 glycans
iPTMnet P01588
GlyGen P01588 5 sites, 190 N-linked glycans (4 sites), 18 O-linked glycans (2 sites)
b.
Glycosylation - Reported Sites with Glycan
Reset Filters
By Amino Acid
By Glycosylation Type
By Mass
By Monosaccharide
By Organism
c.
General
Feature View
3D View
Organism
Names
Motifs
Associated Protein
Glycan Binding Protein
Biosynthetic Enzymes
Subsumption
Biomarkers
Expression
Digital Sequence
Cross References
History
Publications
LOOK AT
Details For Glycan G25392ZR
Back
Add To
DOWNLOAD
General
Sand Box
Related Glycans
GlyTouCan Accession: G25392ZR
Monoisotopic Mass: 4,776.69 Da
Monoisotopic Mass-pMe (Da): 5,911.96 Da
Composition: Hex10 HexNAc9 dHex1 NeuAc4
Byonic™: HexNAc(9)Hex(10)dHex(1)NeuAc(4)
Glycan Type / Glycan Subtype: N-linked / Complex
N-linked / Core-fucosylated
N-linked / Tetraantennary
InChI Key: FRMOAGMUPUTRSA-MOOAQYNDSA-N
d.
PubChem
About
Docs
Submit
Contact
Quick Search PubChem
Advanced Search
Search History
Help
COMPOUND
GlyTouCan:G25392ZR
Cite
Download
CONTENTS
Title and Summary
1 Structures
2 Biologic Description
3 Names and Identifiers
4 Chemical and Physical Properties
5 Related Records
6 Literature
7 Classification
8 Information Sources
PubChem CID 91852997
Structure
2D
Molecular Formula C182H297N13O132
Synonyms RefChem:966256
GlyTouCan:G25392ZR
G25392ZR
CHEBI:149139
CID 91852997
View More...
Molecular Weight 4779 g/mol
Computed by PubChem 2.2 (PubChem release 2025.09.15)
Dates Create: 2015-09-10 Modify: 2026-05-17
Description CID 91852997 is a polysaccharide.
ChEBI
